# Retrograde intramedullary nailing or locked plating for stabilisation of distal femoral fractures? A comparative study of 193 patients

**DOI:** 10.1007/s00590-023-03650-7

**Published:** 2023-08-24

**Authors:** Anthony Howard, A. Myatt, H. Hodgson, H. Naeem, S. Pepple, A. Perumal, M. Panteli, N. Kanakaris, P. V. Giannoudis

**Affiliations:** 1 Leeds Institute of Rheumatic and Musculoskeletal Medicine, University of Leeds, Leeds, UK; 2 Leeds General Infirmary University Hospital, Leeds, UK; 3 NDORMS, Oxford University, Oxford, UK

**Keywords:** Femoral fracture, Plates, Intramedullary nail, Complications

## Abstract

**Purpose:**

The aim of this study was to evaluate the results of distal femoral fracture fixation of two different methods, lateral locking plate (LP) or an Intra-medullary nail (IMN), in patients managed in our institution. More specifically, to assess: (a) if there was a difference in functional outcomes between the LP and IMN groups; (b) whether the rate of complications was different between the two groups.

**Methods:**

Between January 2009 and December 2018 adult patients with distal femoral fractures managed in our unit with either LP or IMN for extra and intra-articular fractures were eligible to participate. Demographic details, fracture type, procedures performed, time to union, complications and functional scores (Oxford Knee Score) were recorded and analysed. The mean follow up was 4 years (12–120 months).

**Results:**

Out of 193 patients who met the inclusion criteria, 93 received an IMN whereas 100 patients were treated with LP. Mean age was 64.2 (18–99) and 70.1 (18–100) for the IMN and LP groups respectively. Overall, the two groups had similar demographics and there was no significant difference in the type of fractures sustained (*p* > 0.05). The Oxford Knee Score was highest for patients fixed with LP, mean 37.3 (6–48, SD 7.3) versus 28.4 (3–48, SD 14.4), (*p* =  < 0.02) compared to the IMN group. In terms of complications, the rate of non-union was higher in the LP group 8.6% versus 4% in those patients treated with an IMN, *p* value < 0.01.

**Conclusion:**

While the rate of non-union was higher in the LP group and the functional results were superior in the plating group.

## Introduction

Distal femoral fractures account for 3–6% of all femoral fractures [1, 2]. There is a bimodal distribution of fracture incidence: younger patients sustaining high-energy injuries and an older group, predominantly women with osteoporosis presenting after a fall from standing height. Lately, a third group is emerging who have sustained periprosthetic fractures around a Total Knee Replacement (TKR), with an incidence of 0.4% after primary TKR [3]. Previous studies are shown in Table 1.

**Table 1:** Previous studies comparing of Retrograde IMN and LP surgical fixation for distal femoral fractures

References	Year	Nos patients	Follow up (mths)	Comparison	Conclusion
Markmiller [18]	2004	32	12	LISS plate v retrograde IMN	No difference between the two group, although the nails had less infections and malunions at 1 year. (Functional score –lysholm)
Hierholzer [21]	2011	118	6	LISS plate v retrograde IMN	Clinical outcomes are dependant on surgical technique rather than implant selection. (Functional score–KOOS)
Gao [16]	2013	36	13–29	Locking plate v retrograde IMN	Union rate was higher in the IMN group Functional score–range of motion
Tornetta [29]	2013	166	12	Locking plate v antegrade IMN	Better functional results (Short Musculoskeletal Functional Assessment) in the IMN (conference proceedings)
Demirtas [15]	2014	28	20–46	Bridge plating v retrograde IMN	In extra- articular fractures the results of union, mal-union, implant failure were similar. (Functional score–Sanders criteria)
Meneghini [19]	2014	91	6–176	Locking plate v retrograde IMN	The rate of non-unions in the IM nail group was 9% compared to 19% non-union/delayed unions in the LP group No functional score
Park [20]	2016	41	24–66	Locking plate v retrograde IMN	IMN group had a higher rate of malunion, no statistical difference between the clinical outcomes of both groups
Gill [17]	2017	42	3–16	Locking plate v retrograde IMN	IMN produced greater operative time and blood loss. The functional and union time between the two groups was not statistically significant different
Ocalan [24]	2019	97	41–130	Locking plate v antegrade IMN	Antegrade IMN achieved significantly better functional outcomes and less non-unions. (Functional score –lysholm)

The clinical need for a comparison has been recognised, and a feasibility study (Trial of Acute Femoral Fracture Fixation (TrAFFix)) has been recently undertaken. FrAFFix found recruitment difficult, and the protocol has been revised [5]. There are a number of studies currently running, which will not complete for a number of years; a RCT running in St Michael’s Hospital, Toronto completing in 2022 [6], is likely to extend further due to COVID 19.

The primary aim of the analysis was to assess whether there was a difference in functional results when treating distal femoral fractures with IMN or LP, as measured through the Oxford Knee Score. The secondary aim was to compare union rates and metal work removal rates between the two cohorts. The hypothesis of this study was that there is no difference in outcomes between these two methods of treatment.

## Patients and methods

The electronic records of our institution were interrogated to identify patients treated with Lateral Locking Plate (LP) or Intramedulary Nail (IMN) for extra and intra-articular fractures of the distal femur over a 10-year period (2009–2018). Inclusion criteria were patients with unilateral injury, age > 18, minimum follow up of 12 months, intra and extra-articular fractures within distal 1/3 of the femoral shaft) or a peri-prosthetic fracture. Exclusion criteria were open fractures, pathological fractures and patients with distal femoral fractures that were managed with acute total knee replacement.

The Arbeitsgemein-schaft für Osteosynthesefragen/Orthopaedic Trauma Association (AO/OTA) classification [7] was used to define what we defined as distal femoral fractures which includes all fractures falling with AO distal femur (Category 32 and 33 excluding 33B and 33C). Two members of the research team classified the fractures and assessed fracture healing radiographically, with any disagreement resolved by the senior author. Such details were documented and entered in a computerised database as patient demographics, Charlson co-morbidities index [8], other associated injuries, mechanism of injury, method of reduction and fixation, blood loss, complications, time to union, length of hospital stay and reinterventions.

### Treatment protocol

On presentation, all patients were initially managed according to the principles of Advanced Trauma Life Support protocol [9]. Once optimised, patients were taken to theatre and were operated under regional or general anaesthesia. All patients were operated on by a Consultant or under their direct supervision.

At induction prophylactic intravenous antibiotics were given (gentamycin and flucloxacillin). All procedures were carried out with the patient in supine position on a radiolucent table (OSI). Standard retrograde IM nailing technique was used with reaming performed at least 1.5mm greater that the selected nail diameter. LP was carried out utilising a lateral incision with the plate inserted either using a minimal invasive plate osteosynthesis (MIPO) or an open approach as it was indicated. After surgery, both patient groups were permitted to weight bear as tolerated. All patients received thromboprophylaxis (Tinzaparin 4,500IU) for a period of 6 weeks. Following discharge from the hospital, all patients were followed up in the orthopaedic outpatient clinic at 6 and 12 weeks, then at 6, 9, 12 months or longer as it was indicated for both clinical and radiological review. All the patient images were assessed using the RUST score [10].

The Oxford Knee Score [11, 12] assesses the functional results of treatment. This scoring is derived from 12 questions, producing a score between 0 and 48, with the high values indicating a normal knee joint. The score was completed at the last follow up or over a telephone interview in elderly individuals, this provided the opportunity for assistance in completing the questionnaire which has been shown to improve the reliability of this scoring system [12]. The minimum follow-up was 12 months (range 1–10 years). Serial radiographs obtained through out the treatment course were assessed and union was defined as bridging callus formation on three or more cortices [13].

### Statistical analysis

The IBM SPSS Statistics 25.0 program (IBM SPSS Statistics for Mac, version 25.0, Armonk, NY: IBM Corp.) and the SAS 9.3 program were used to perform all statically analyses for the current study. All values of *p* < 0.05 were considered to be significant. Descriptive statistics were used as follows: mean, range, standard deviation (SD). Quantitative variables were evaluated with the Mann–Whitney-U test. The Fisher’s exact probability test and the Person Chi-Square test were used to determine if there was any correlation among categorical variables.

### Source of funding/ethics

No funding was obtained. The institutional review board of the hospital approved the study, (IRB number 8596).

## Results

**Fig. 1:**
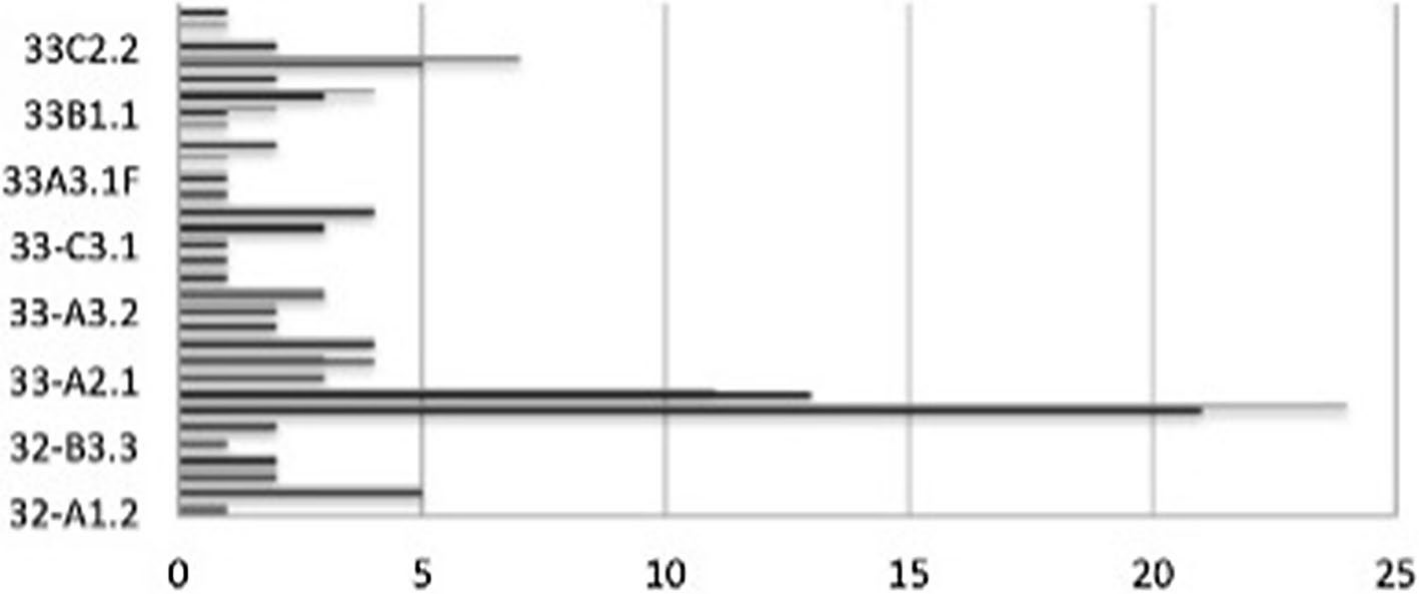
Fracture profile (AO classification) of the locking plate (black) and intramedullary nails (grey)

**Fig. 2:**
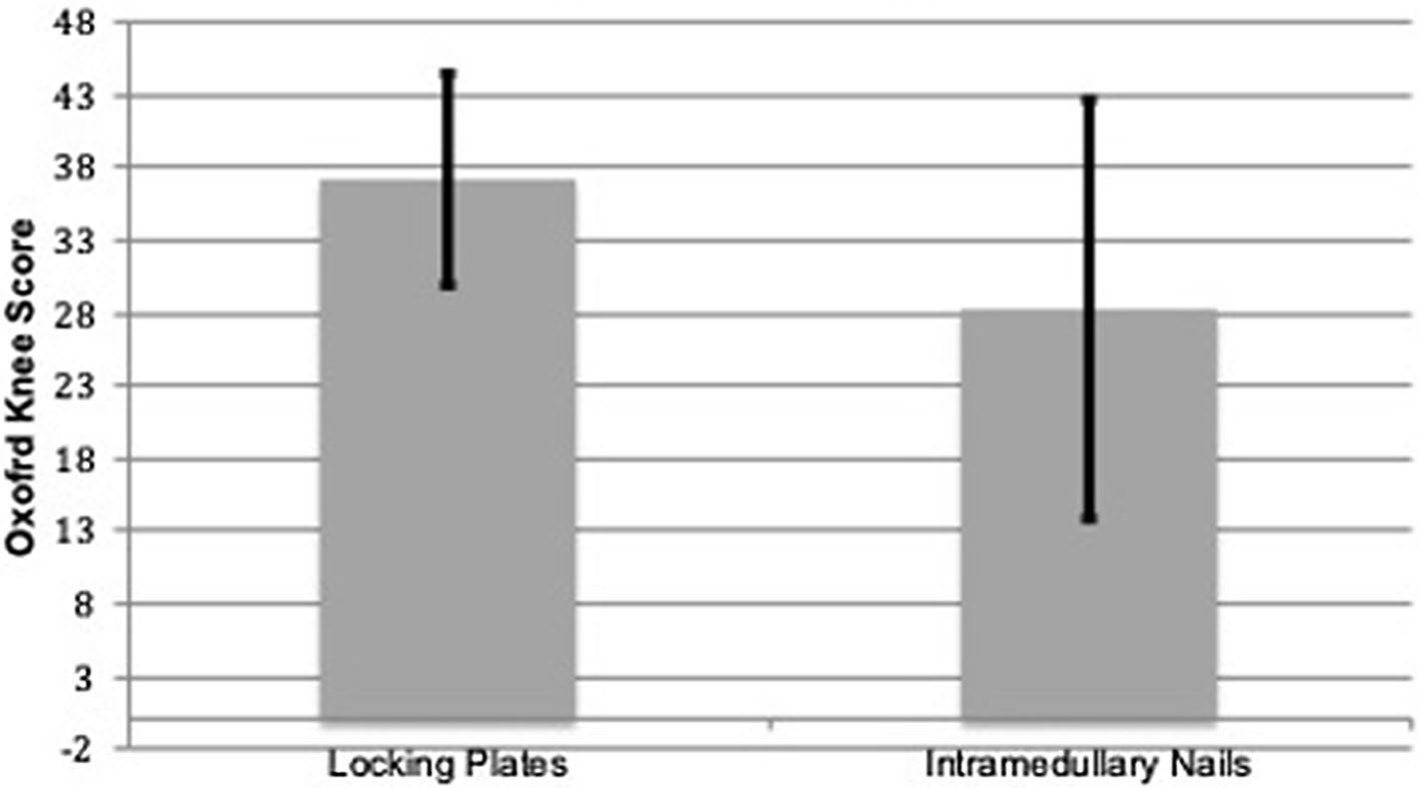
Mean Oxford Knee Score (+ /1) SD

Overall, 193 patients met the inclusion criteria (93 receiving an IMN and 100 treated with LP). Figure 1 shows that there was no significant difference in the type of fracture between the two treatment groups (*p* > 0.05). The two groups had similar demographics with mean age of 64.2 (16–99) and 70.1 (19–100) for the IMN and LP groups respectively (*p* > 0.05), Fig. 2. The ratio of male/female was 1:3.0 in the IMN group and 1:2.8 in the LP group. The mean follow-up for LP group was 4.0 years (1.0–8.6) and for the IMN 4.1 years (1.0–9.5).

The Charlson Comorbidity Index for both patient groups was not significantly different (*p* > 0.05), with a mean score of 3.6 (0–10) for those patient receiving IMN and a mean score of 3.9 (0–7) for patients treated with a LP. There was no statistically significant difference in the length of hospital stay, Table 2, and time until the patient was discharged from clinic following satisfactory completion of their treatment.

**Table 2:** Patient and treatment related demographics

	Intramedullary nails (mean)	Locking plates (mean)
Age	64.2 (18–99, SD 23.4)	70.1 (19–100, SD 19.6)
Sex (m/f)	1:3.03	1:2.82
Length of hospital stay (days)	26.2 (6–128, SD 25.7)	26.1 (8–362, SD 42.1)
Length of clinical supervision (years)	1.1 (1.0–5.0, SD 1.1)	1.1 (1.0–5.0, SD 1.1)
Associated injuries/isolated injury	28/65	16/84

### Functional results

The OKS score, Fig. 2, was highest for those patients fixed with LP, mean 37.3 (6–48, SD 7.3) while for the IMN patient group the mean was 28.4 (3–48, SD 14.4). The data were not normally distributed, tested by Kolmugorov-Smirnov test, and the Mann–Whitney U test was deployed. The OKS of the IMN and the LP was significantly different (*p* < 0.01). The patient Oxford Knee Score was divided into different lengths of follow up, Table 3. When separated the mean OKS of the LP patient group remained higher than IMN. The majority of length of follow-up categories (1–2 years, 6–8 years, 8–10 years) were statistically significant (*p* < 0.02).

**Table 3:** Functional results measured through the Oxford Knee Score categorised by length of patient follow-up

Years	Number of patients	Intramedullary nails Oxford Knee Score (mean)	Locking plates Oxford Knee Score (mean)	*p* value
1–2	24	22.1 (13–36)	38 (23–47)	< 0.01
2–4	46	28.8 (13–48)	37.6 (22–47)	0.62
4–6	72	34.1 (10–48)	37.8 (22–47)	0.91
6–8	14	19.8 (11–31)	37.8 (29–43)	0.02
8–10	36	39 (30–48)	41.5 (40–43)	< 0.01

**Fig. 3:**
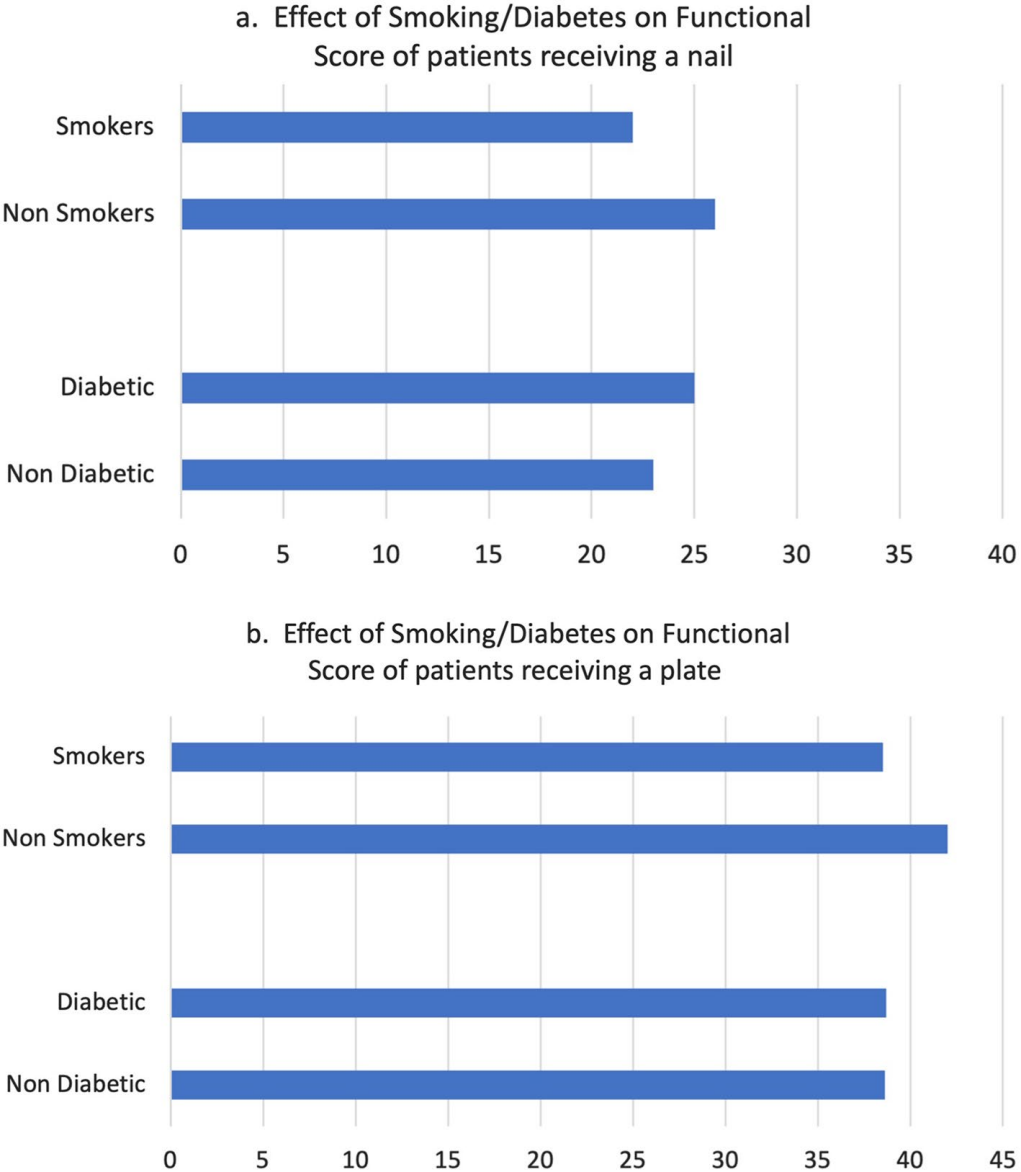
The effect of smoking/diabetes on the functional scores of IMN patients **a** and LP patients

Interestingly, the patient sample studied contained patients who had a Total knee replacement in the effected side, IMN group (*n* = 6) and the LP group (*n* = 16). When these were removed from the groups, the mean OKS in the LP group was 38.4 (23–47) and in the IMN group 24.2 (3–46). Comparison of the OKS in both groups showed a significant difference (*p* < 0.01). A similar picture occurred when the patient groups were divided into intra and extra-articular fracture and the two fixation devices compared, (*p* < 0.05). It is well recognise that smoking and diabetes influence the patient’s functional results after long bone fractures [14]. However, these influences on functional recovery showed an impact only in the LP group, Fig. 3.

### Complications

The time to union, as defined above, was quicker in the IMN group (119.4) days (25–343), compared to the LP group 174.8 days (44–332), (*p* < 0.01). Using a RUST score of 12 as the end point for the IMN group this was achieved in 244.8 days (64–635) and for the LP group in 277.5 days (41–546) (*p* < 0.02). This is an important metric as it is associated with less pain and could enable a shorter absence from work.

The rate of non-union was higher in the LP group 8.6% versus 4% in those patients treated with an IMN, *p* values < 0.01. Where non-union occurred, IMN group was treated with exchange nailing, whilst the non-unions in the LP were treated with a combination of bone grafting and revision to an IMN. In 6% of the IMN group, screws needed to be removed due to soft tissue irritation, which was comparatively high compared to the LP group (1.1%), (*p* < 0.05). Respiratory infections/DVT were 11% in the LP group and 10.8% in the IMN group.

## Discussion

This study represents the most extensive retrospective study ever undertaken comparing IMN and LP of distal femoral fractures, including 193 patients. There are currently 9 studies retrospectively comparing LP and IMN, [15–21], Table 1. All but one of these studies [19] includes functional scores, ranging from assessment of range of motion, to the KOOS [22] or Lysholm Score [23]. The functional scores between the two techniques have been largely equivocal, with only two studies [24] [18] concluding that function in the IMN group was statically significantly superior.

Interestingly, several studies have evaluated the IMN [4, 25] and LP [26] fixation method in isolation, or hybrids of the two systems in biomechanical studies [27]. Other studies have considered variations in surgical techniques of both methods of fixation [28, 29], and different LP [26]. A recent Cochrane Review (2015), concluded that on the current evidence there was insufficient evidence to inform clinical practice [30]. A more recent meta-analysis of both surgical techniques for periprosthetic fractures found there was no statistically significance differences in union rate, union time and complication rates [31]. In contrast, Herrera DA et al. in their systematic review reported less rates of non-union and revision surgery in the IMN group [32].

Distal femoral fractures are potential life-changing injuries. Making the surgical choice on the optimum fixation technique is critical to mitigating against functional deficiency and complications. Both LP and IMN are recognised as effective fixation techniques in distal femoral fractures, with the choice often based on anecdotal or poor-quality evidence and surgeon preference.

The demographics (Table 2), and the fracture configuration (Fig. 1), of the two groups, there is a higher proportion of patients with associated injuries in the IMN group (4.13%.) compared to the LP group (19%). The study was strengthened by inclusion of patient co-morbidities overcoming short-comings of a retrospective analysis [33]. Functional recovery and rate of complications are the principal surgical concerns in long bone fixation.

Assessing our primary research question, we used the OKS as a measure of functional outcome between patients who had received LP and IMN. Returning the patient to the pre-accident level of function is one of the primary goals of acute reconstruction surgery. Overall, patients treated with LP had a better functional recovery, statistically significantly so, with a mean score of 37.3 compared to the IMN patient group mean score 28.4. Interestingly, the LP patient group outperformed the IMN one even when patients were divided into different time scales from the point of the original surgery. However, it has to be accepted that the OKS is a soft marker of functional recovery and that pre-accident OKS were not available.

When the potentially confounding influences (intra versus extra-articular fracture/peri-prosthetic fractures) was removed from the different treatment groups, the LP retained their function advantage over the nails. Smoking and diabetes influence the patient’s functional results after long bone fractures but showed an impact only in the LP group, Fig. 3. The reason for this is unclear but one can speculate that the retention of the soft tissue envelope and the less violation of the intracapsular structures could be the reasons associated with this finding.

The data from our study support the view that LP performed functionally superior to the IMNs, which contradicts previous small studies [18, 24]. However, this needs to be approached with caution, as it has been reported that both techniques rely on good technical surgical skills to achieve good functional outcomes [21]. Thus, a surgeon who is more technically skilled in IMN should consider changing the type of fixation used with the benefit of input from surgical colleagues undertaking higher volumes of LP fixation. On the data collected in this study we can explain the causal of the apparent difference in outcomes, and will form the basis of future work.

The secondary outcomes, of the rates of unions and need to remove metal work were assessed. The principle difference between the LP and IMN techniques is that the former being a load bearing device and pending how is used facilitates fracture union by either primary or secondary bone healing. In contrast, IM nailing (a mainly load sharing device) promotes union by secondary bone healing [14]. When LP are not used appropriately (ie over-ridged plate configuration) this can lead to non-union as it was reported by Wang MT et al. in a systematic review of the literature [34]. The LP group in this study had double the number of non-unions compared to the IMN group. Moreover, the time to union was longer in the LP compared to IMN group. This can be explained by the biomechanical advantage of the IMN, which is a load-sharing device, enabling early mobilisation, which to a certain extent increases the factors that facilitate union, bone contact and micromotion at the fracture site. Both groups needed further surgical intervention either exchange nailing or swapping from LP to IMN with bone grafting to promote healing. However, this needs to be counter-balanced against 6% of patients with IMN required screws to be removed due to pain related to soft tissue irritation. A Comparison of the two groups showed no statically significant difference between the length of hospital stay and length of clinical follow up (*p* < 0.05).

### Study limitations

This study has some limitations. Firstly, is of retrospective nature with no randomisation. However, the good sample size, long selection period and large number of operating surgeons, militates against this weakness. The method of fixation was not blinded to the patients. The retrospective nature means we have no short-term functional scores prior to 12 month post-operatively. These would be important in comparing the ability to return to work earlier. Further, the proportion of associated injuries is higher in the IMN group compared to the LP group. There is a wide selection of types of distal femoral fractures and in the LP group, although the majority were treated with a LISS plate, other types of plates were employed.

Secondly, different surgeons with different learning curves performed the procedures. Thirdly, the state of the bone fragility, which could have influenced the results of fixation was not assessed in this study. Furthermore, it has to be accepted that the Oxford Knee Score makes no provision for co-morbidities, given we know that a proportion of both patient groups possess a number of co-morbidities this should be borne in mind when considering the results [12]. If another outcome measure had been selected, then different results may have been found. Finally, assessment of fracture healing through radiographs has been criticised [35] and the time interval was dictated by normal treatment protocols rather than research assessment.

Strengths of the study include the prospective gathering of Oxford Knee Score of patients within the study. This cannot overcome the limitation of a retrospective study but provide clinically useful functional scores in comparing the two fracture fixation techniques. In addition, there has been an attempt to militate against the limitations of any retrospective review by seeking to use factors such as the Charlson Index to reduce the number of confounding factors.

In conclusion, this is the largest retrospective study undertaken comparing IMN and LP fixation of distal femoral fractures. Against the primary research question, the functional results of patients are superior in the LP group compared the IMN group, when assessed in post-operative time frames from 1 to 10 years. This functional advantage has to be weighed up against the increased risk of non-union in the LP. There is clearly a need for a well-designed randomised clinical trial, and although some have started, it will be a while before the results are likely to be available.
